# Evolution of Tertiary Structure of Viral RNA Dependent Polymerases

**DOI:** 10.1371/journal.pone.0096070

**Published:** 2014-05-09

**Authors:** Jiří Černý, Barbora Černá Bolfíková, James J. Valdés, Libor Grubhoffer, Daniel Růžek

**Affiliations:** 1 Institute of Parasitology, Biology Centre of the Academy of Sciences of the Czech Republic, České Budějovice, Czech Republic; 2 Faculty of Science, University of South Bohemia in České Budějovice, České Budějovice, Czech Republic; 3 Faculty of Tropical AgriSciences, Czech University of Life Sciences Prague, Prague, Czech Republic; 4 Veterinary Research Institute, Brno, Czech Republic; Meharry Medical College, United States of America

## Abstract

Viral RNA dependent polymerases (vRdPs) are present in all RNA viruses; unfortunately, their sequence similarity is too low for phylogenetic studies. Nevertheless, vRdP protein structures are remarkably conserved. In this study, we used the structural similarity of vRdPs to reconstruct their evolutionary history. The major strength of this work is in unifying sequence and structural data into a single quantitative phylogenetic analysis, using powerful a Bayesian approach.

The resulting phylogram of vRdPs demonstrates that RNA-dependent DNA polymerases (RdDPs) of viruses within *Retroviridae* family cluster in a clearly separated group of vRdPs, while RNA-dependent RNA polymerases (RdRPs) of dsRNA and +ssRNA viruses are mixed together. This evidence supports the hypothesis that RdRPs replicating +ssRNA viruses evolved multiple times from RdRPs replicating +dsRNA viruses, and *vice versa*. Moreover, our phylogram may be presented as a scheme for RNA virus evolution. The results are in concordance with the actual concept of RNA virus evolution. Finally, the methods used in our work provide a new direction for studying ancient virus evolution.

## Introduction

RNA viruses evolve rapidly. Since viral RNA-dependent polymerases (vRdP) miss the proofreading activity they produce a high percentage of mutated variants [1]. These variants face a strong evolutionary pressure by the host immune system and a highly competitive environment between relative viruses [2]. These factors lead to a rapid diversification in the primary structure of all viral genes and proteins, and a swift establishment of new virus strains [3]–[5].

Despite these fast changes in the sequences of viral proteins, functions that are crucial for efficient virus reproduction must be preserved [6]. Therefore, proteins involved in important steps of the virus life cycle accumulate mutations slower and preserve a higher degree of conservation [6]. The most conserved proteins among RNA viruses are polymerases, helicases, proteases and methyltransferases [7].

Contrary to the primary structure, the tertiary structure of most proteins sharing a common evolutionary origin remains conserved [8], [9]. The most conserved part of the protein is usually the core structure essential for protein function. The core is often surrounded by less conserved structures modifying the protein function. Changes in these additional structures often lead to minor changes in protein character (e. g., different substrate specificity), but the major protein function remains unchanged.

Morphological description of protein structure can help in reconstructing protein evolutionary history. In this approach, protein structural features are encoded in a character matrix where the rows describe the individual proteins and the columns describe the individual features. This is similar to the approach used for reconstructing the evolutionary relations among fossil species [10]. Morphological data can also be coupled with sequence data to enforce the incoming information [11], [12]. This approach may also be applied to proteins. For example, mixed morphological and sequence data were used to reconstruct the evolution of aminoacyl tRNA synthetases class I [13] and the protein kinase-like superfamily [14].

Among all viral proteins, vRdPs display the highest degree of conservation. Genes coding for vRdPs were found in all non-satellite RNA viruses and RNA viruses reproducing via a DNA intermediate [15]. All vRdPs contain seven typical sequence motifs (G, F, A, B, C, D and E) [16], [17] that incorporate conserved amino acid residues crucial for polymerase function [18], [19].

Moreover, vRdPs share remarkable structural homology. The protein structural fold resembles a right hand with subdomains termed fingers, palm and thumb [20]–[23]. The palm subdomain is structurally well conserved among all vRdPs. Finger and thumb subdomains are more variable, but they can be fully aligned only among RNA-dependent RNA polymerases (RdRPs) of +ssRNA viruses [21]. For most vRdPs, the finger, palm and thumb subdomains accommodate seven conserved structural motifs (homomorphs), each bearing one of the conserved sequence motif described before [24].

All vRdPs evolved from one common ancestral protein [16], [20]. In the past, sequence similarity among vRdPs was used in attempts to reconstruct RNA virus evolutionary history [7], [16], [25]–[31]. Unfortunately, this sequence similarity was shown to be too low to produce an accurate sequence alignment for further phylogenetic analysis [32].

In our current work, we used the structural similarity of vRdPs to reconstruct their evolutionary history. We used the similarities of vRdPs protein structures to produce a highly accurate structure based sequence alignment for our subsequent studies. Moreover, we picked 21 biochemical and structural features of each polymerase and encoded them into the matrix that was used in a phylogenetic analysis to particularize results obtained from structure based sequence alignment analysis. In our phylogenetic analysis, we used Bayesian clustering algorithms, which are ideal for reconstruction of complicated phylogenetic relationships. The resulting phylogenetic tree describing the evolution of vRdPs has high statistical support for most branches. As vRdPs are the only universal gene in all RNA viruses, our phylogenetic tree can be understood as a scheme of RNA virus evolution.

## Materials and Methods

### Selection of vRdPs for further phylogenetic studies

To find structurally homologous vRdPs, we employed the DALI server [33] using the structure of Dengue virus type 3 (DENV3) RdRP as a query (PDB number 2J7W-A). The program was run under the default conditions. DALI server automatically screens the PDB database to select structurally homologous proteins and lists them according to a decreasing Z-score, a quantitative expression of protein structure similarity [33]. Only protein structures having similarity Z score higher than 2 were taken in account since hits with lower Z-score are most likely incidental hits. The vRdPs were selected among the listed protein structures. They were assigned to the individual virus species classified into genera and families according to the actual ICTV virus taxonomy [34]. Representative structures were selected using the following criteria: (1) Maximally two polymerases from two different viruses were selected from one genus (the exception was four viruses from genus *Enterovirus*). (2) Structures with bound substrate, substrate analogue and/or template nucleic acid were favored. (3) High resolution structures were preferred. (4) Structures without any mutation were favored. As polymerases are very active enzymes changing their topology in response to many external stimuli (bound template/nucleotide/product, actual step of polymerization cycle, etc.), the criteria for structure selection was set up to select polymerase structures under identical conditions.

The same process described above was done using three structures with the lowest structure homology to 2J7W-A as queries using the DALI sever: 3V81-C (human immunodeficiency virus 1 - HIV1), 2R7W-A (simian rotavirus - SRV) and 2PUS-A (infectious bursal disease virus - IBDV). Sets of structures selected in these three runs were compared with the first set to insure no adequate structures were missed.

### Construction of structure superposition and structure based sequence alignment

Structures of selected vRdPs were superimposed using the DALI server multiple structural alignment tool [33]. DALI created structure based sequence alignment was validated and improved using the default settings in T-Coffee Expresso [35]. The resulting alignment was verified by comparison with previously published vRdP alignments [17], [24], [31], [36], [37].

The structure based sequence alignment was analyzed using the JOY server under the default conditions [38]. JOY is a program used for annotation of protein sequence alignments with 3D structural features. It is necessary in understanding the conservation of specific amino acid residues in a specific environment. JOY contains various algorithms such as DSSP [39] used for secondary structure classification. Sequence consensus and sequence conservation were calculated in Chimera implemented algorithms [40], [41].

### Analysis of the vRdPs structural similarities between vRdPs

Analysis of conserved amino acid residues and sequence motifs in the structural based sequence alignment as well as presence/absence of conserved structural features was done manually according to criteria previously used in describing vRdPs [20], [24], [42]. Comparative results were encoded into a 21-column character matrix where each column represents a single selected character typical of some but not all vRdPs. The matrix row represents each evaluated polymerase. Structural characters were coded to MrBayes as standard data (0–9). These characters were set as unordered allowing them to move from one state to another (character designated “0” can change to “2” without passing “1”).

### Construction of phylogenetic tree

Best fitting model of amino acid substitutions was tested in PROTTEST 2.4 [43] under the Akaike information criterion [44] and the Bayesian information criterion [45]. As results of the two tests were not consistent, we decided to use the most complex model, the general time reversible (GTR) model with a proportion of invariable sites and a gamma-shaped distribution of rates across sites [46], [47]. Bayesian phylogenetic analysis was performed using MrBayes v3.1.2 [48]. Bayesian analysis consisted of two runs with four chains (one cold and three heated), and was run for 10 million generations sampled every 100 generations. The first 25% of samples were discarded as a burning period. Although the average standard deviation of split frequencies was much lower than 0.01, convergence of runs and chains was verified using the AWTY [49]. Analysis was run for sequence data alone and for mixed data (sequence alignment and structural character matrix) with equal settings for analysis.

## Results

### Formation of representative set of vRdPs

The DALI server queried using the Dengue virus RdRP (2J7W-A) found 745 hits with structure similarity Z-score 2 or higher. Using the criteria described in the Material and methods section, we selected 21 vRdPs protein structures among these hits. In our subsequent query, no additional protein structures were selected from 844, 743 and 575 hits identified using 3V81-C (HIV1), 2R7W-A (SRV), and 2PUS-A (IBDV).

To ensure we did not miss any relevant structure, we browsed the PDB [50] using names of all RNA virus genera listed in the ICTV database. No additional structures were found. A preliminary notice was found about the successful crystallization of *Thosea asigna* virus RdRP (genus *Permutotetravirus*, family *Permutotetraviridae*), but the structure has not yet been published [51].

The final list included 22 vRdPs from 22 virus species in 17 virus genera and 8 virus families (see Table 1 for details). All viral families were classified in the Baltimore classes III (double stranded RNA viruses), IV (positive sense single stranded RNA viruses), and VI (Positive-sense single-stranded RNA viruses that replicate through a DNA intermediate). No polymerases of any virus classified in Baltimore class V (negative sense single stranded RNA viruses) were identified, since there was no known protein structure of any RNA dependent RNA polymerase for these viruses.

**Table 1:** The list of selected vRdPs.

Baltimore class	family	genus	virus	abbre-viation	viral RNA dependent polymerase				
					PDB	str.	res. [Å]	cocrystallized molecules	citation
+ssRNA viruses	*Caliciviriade*	*Lagovirus*	Rabbit hemorrhagic disease virus	RHEV	1KHV	B	2,5	Lu^2+^	[90]
		*Norovirus*	Murine norovirus	MuNORV1	3UQS	A	2	SO_4_^2−^	[91]
			Norovirus	NORV	3BSO	A	1,74	Mg^2+^, CTP, RNA	[92]
		*Sapovirus*	Sapporo virus	SappV	2CKW	A	2,3		[93]
	*Flaviviridae*	*Flavivirus*	Dengue virus 3	DENV3	2J7W	A	2,6	Zn^2+^, GTP	[94]
			Japanese encephalitis virus	JEV	4K6M	A	2,6	SAH, SO_4_^2−^, Zn^2+^	[95]
		*Hepacivirus*	Hepatitis C virus 1	HCV1	1NB6	A	2,6	Mn^2+^, UTP	[96]
		*Pestivirus*	Bovine viral diarrhea virus	BVDV1	1S49	A	3	GTP	[97]
	*Leviviridae*	*Allolevivirus*	Enterobacterio phage Qβ	Qβ	3AVX	A	2,41	Ca2+, 3′dGTP, RNA	[98]
	*Picornaviridae*	*Aphthovirus*	Foot and mouth disease virus	FMDV	2E9Z	A	3	Mg2+, UTP, PP_i_, RNA	[99]
		*Enterovirus*	Humane rhinovirus 16 A	HuRV16A	1XR7	A	2,3		[100]
			Coxsackie virus B3	CoxVB3	3CDW	A	2,5	PP_i_	[101]
			Humane rhinovirus 1B	HuRV1B	1XR6	A	2,5	K^+^	[100]
			Poliovirus 1	PolV	3OLB	A	2,41	Zn2+, ddCTP, RNA	[42]
ds RNA viruses	*Birnaviridae*	*Aquabirnavirus*	Infectious pancreatic necrosis virus	IPNV	2YI9	A	2,2	Mg^2+^	[102]
		*Avibirnavirus*	Infectious bursal disease virus	IBDV	2PUS	A	2,4		[103]
	*Cystoviridae*	*Cystovirus*	Pseudomonas phage phi6	Φ6	1HI0	P	3	Mn^2+^, Mg^2+^, GTP, DNA	[62]
	*Reoviridae*	*Orthoreovirus*	Mammalian orthoreovirus 3	MORV3	1N35	A	2,5	Mn2+, 3′dCTP, RNA	[104]
		*Rotavirus*	Simian rotavirus Sa11	SRV	2R7W	A	2,6	GTP, RNA	[105]
Reverse tran- scribing viruses	*Retroviridae*	*Gammaretrovirus*	Moloney murine leukemia virus	MoMLV	1RW3	A	3		[106]
		*Lentivirus*	Human immunodeficiency virus 2	HIV2	1MU2	A	2,35	SO_4_^2−^	[107]
			Human immunodeficiency virus 1	HIV1	3V81	C	2,85	nepavirine, DNA	[108]

The vRdPs selected as described in Material and methods were assigned to individual viral species, genera, families and Baltimore groups. For each individual vRdP its PDB code (PDB), used protein strand (column str.), resolution (column res.) and cofactor, substrate, template, product molecules (column co-crystallized molecules) are listed.

### Structure superposition of vRdPs

The vRdPs from our collection represents a wide range of proteins that are different in protein size and other parameters (see Table 1). Many of them bear additional domains with non-polymerase activities that are conserved only among closely related proteins. These domains were not taken into account for subsequent analysis.

**Figure 1:**
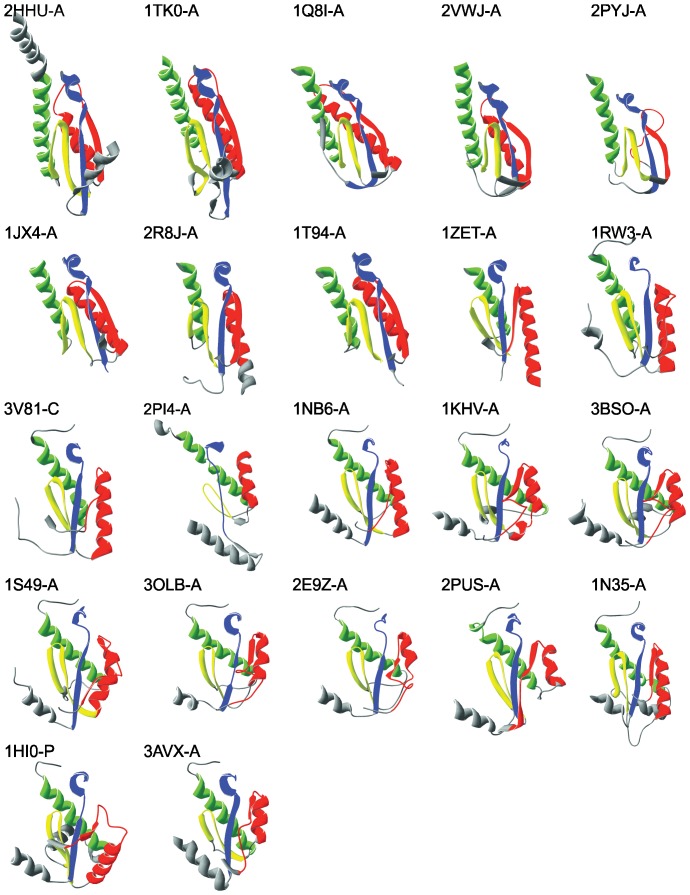
Protein structures of selected vRdPs representatives. Nine representatives of the selected vRdPs were chosen. Their structures are shown as a ribbon diagram. All molecules are oriented in the same orientation with finger subdomain on the left, the palm on the bottom and the thumb on the right. The catalytic site is positioned in the centre of each molecule and in some protein structures it is enclosed by the finger tips located at the top of each protein structure. Conserved protein structures typical of vRdPs (homomorphs) are highlighted by colours: violet (hmG), dark blue (hmF), dark green (hmA), light green (hmB), yellow (hmC), orange (hmD) red (hmE), and pink (hmH). Molecular rendering in this figure were created with Swiss PDB Viewer.

Primary and tertiary structures of domains bearing polymerase activity are similar in all selected proteins. Subdomains finger (F), palm (P), and thumb (T) are collinearly arranged in all vRdPs succeeding always as F1-P1-F2-P2-T from N- to C-terminus (see Figure S1 for details) [20]–[23]. Polymerase domains of selected vRdPs were superpositioned and structures typical for each of the selected viral families are highlighted in Figure 1 (for schematic structure of all vRdPs see Figure S2). Structural superposition shows a conserved architecture of vRdP subdomains and the seven conserved structural homomorphs previously described [24] are clearly visible.

An additional eighth structural helix-turn-helix motif was observed in the thumb subdomain, we call homomorph H (hmH). Despite the poorly conserved sequence of homomorph H, the structural motif is well conserved in all vRdPs (see Figure 1). To characterize its conservativeness, we calculated its RMSD among all vRdPs and compared it with the RMSD of homomorph D (hmD) that is similar in size. Results showed that hmH is as conserved as the well-established hmD (see Table S1 for further details).

### Structural similarities among vRdPs

The structure similarity Z-score was calculated for all polymerase couples (see Table 2) showing extremely high protein structure similarities among vRdPs from viruses classified into one viral genus (see genus *Enterovirus* as the best example). The similarities among the vRdPs of viruses classified in the same family are slightly lower, but still very high (see family *Picornaviridae* as the best example). RdRPs of all +ssRNA viruses (except enterobacteriophage Qβ - Qβ) form a cluster of relatively highly similar structures, while structures of pseudomonas phage Φ6 (Φ6), Qβ and *Birnaviridae* RdRPs are moderately similar, and structures of reoviral RdRPs and retroviral RdDPs are similar only distantly to RdRPs of +ssRNA virus (see Table 2 for details).

**Table 2:** Comparison of structure similarity Z-score of all vRdPs.

		DENV	JEV	BVDV1	HCV1	PolV1	HuRV16	HuRV1B	CoxVB3	FMDV	NORV	MuNORV1	RHEV	SappV	Φ6	Qβ	IBDV	IPNV	SRV	MORV3	HIV1	HIV2
		2J7W-A	4K6M-A	1S49-A	1NB6-A	3OLB-A	1XR7-A	1XR6-A	3CDW-A	2E9Z-A	3BSO-A	3UQS-A	1KHV-B	2CKW-A	1HI0-P	3AVX-A	2PUS-A	2YI9-A	2R7W-A	1N35-A	3V81-C	1MU2-A
JEV	4K6M-A	42,9	-	-	-	-	-	-	-	-	-	-	-	-	-	-	-	-	-	-	-	-
BVDV1	1S49-A	22,8	21,7	-	-	-	-	-	-	-	-	-	-	-	-	-	-	-	-	-	-	-
HCV1	1NB6-A	20,5	17,4	27,4	-	-	-	-	-	-	-	-	-	-	-	-	-	-	-	-	-	-
PolV1	3OLB-A	18,1	16,8	25,3	21,5	-	-	-	-	-	-	-	-	-	-	-	-	-	-	-	-	-
HuRV16	1XR7-A	18,2	16,6	25,1	20,9	52,4	-	-	-	-	-	-	-	-	-	-	-	-	-	-	-	-
HuRV1B	1XR6-A	18,0	16,5	24,8	20,7	52,2	56,7	-	-	-	-	-	-	-	-	-	-	-	-	-	-	-
CoxVB3	3CDW-A	18,0	16,3	25,2	21,0	53,1	52,4	53,1	-	-	-	-	-	-	-	-	-	-	-	-	-	-
FMDV	2E9Z-A	19,2	17,2	26,5	21,6	41,5	41,3	41,0	41,6	-	-	-	-	-	-	-	-	-	-	-	-	-
NORV	3BSO-A	20,5	17,5	27,1	23,8	32,0	32,3	38,1	31,8	32,4	-	-	-	-	-	-	-	-	-	-	-	-
MuNORV1	3UQS-A	20,9	17,7	28,0	25,2	31,1	31,5	31,2	31,4	32,2	51,0	-	-	-	-	-	-	-	-	-	-	-
RHEV	1KHV-B	18,7	17,9	27,4	24,3	32,4	33,0	32,9	33,0	32,4	39,3	42,7	-	-	-	-	-	-	-	-	-	-
SappV	2CKW-A	17,5	15,0	24,7	20,6	30,4	30,8	30,8	30,9	30,8	39,1	39,4	43,9	-	-	-	-	-	-	-	-	-
Φ6	1HI0-P	14,8	10,6	4,1	16,4	17,2	17,0	16,9	17,7	15,7	18,5	19,1	17,7	14,1	-	-	-	-	-	-	-	-
Qβ	3AVX-A	11,1	7,7	14,8	14,1	14,0	13,5	13,6	14,5	13,8	13,2	14,4	14,9	12,6	12,3	-	-	-	-	-	-	-
IBDV	2PUS-A	8,4	6,6	10,7	9,5	12,1	12,1	11,9	12,6	12,9	13,4	13,3	12,6	12,9	9,5	6,0	-	-	-	-	-	-
IPNV	2YI9-A	9,8	6,7	13,9	12,9	12,4	12,3	12,1	13,0	13,5	15,5	14,2	14,0	13,2	10,7	7,7	42,5	-	-	-	-	-
SRV	2R7W-A	8,9	9,0	10,2	10,5	9,7	9,4	8,3	8,4	9,3	9,4	9,1	10,4	8,5	9,9	7,8	4,6	4,6	-	-	-	-
MORV3	1N35-A	6,5	4,0	10,3	7,6	7,8	7,3	7,1	7,8	8,1	7,9	7,9	8,1	8,0	8,4	8,0	6,5	6,6	15,4	-	-	-
HIV1	3V81-C	4,7	1,6	6,3	6,5	5,4	5,5	4,9	4,8	5,3	5,5	5,7	5,7	4,9	3,8	5,8	2,8	2,3	4,0	5,9	-	-
HIV2	1MU2-A	5,4	4,0	7,9	7,4	6,2	6,6	6,8	6,9	6,1	7,6	7,9	6,5	7,4	5,5	7,7	3,6	4,3	4,6	5,1	28,5	-
MoMLV	1RW3-A	4,7	3,4	7,9	6,2	7,2	7,4	7,0	6,8	6,0	7,6	6,8	7,5	7,4	4,9	6,2	2,6	3,0	4,0	3,9	18,2	20,7

Individual vRdP structures are introduced by a PBD code-strain and they are assigned to a virus species. Note that structure similarity Z-score is high among vRdPs originating from viruses classified in the same genus (see genus *Enterovirus* (written in bold) as the best example). Structural similarity is somewhat lower but still high among vRdPs from viruses classified in the same family (see family *Picornaviridae* (written in italic) as the best example). Structural similarity of vRdPs from viruses classified in different families is significantly lower and is decreasing with excepted phylogenetic relationship. Compare all other families to family *Picornaviridae*.

We also quantified 21 attributes previously used for vRdPs description and encoded them into a 21-column character matrix (see Table 3). Features were selected and quantified manually according to criteria previously used for describing vRdPs [20], [24], [42] and are included in the Text S1.

**Table 3:** Matrix describing individual features used in phylogenetic analysis of vRdPs.

Virus	Family	Genus	PDB ID	Chain	Features																				
					A	B	C	D	E	F	G	H	I	J	K	L	M	N	O	P	Q	R	S	T	U
DENV3	*Flaviviridae*	*Flavivirus*	2J7W	A	0	0	0	0	0	0	N	1	0	0	0	0	2	0	0	0	0	0	0	0	1
JEV	*Flaviviridae*	*Flavivirus*	4K6M	A	0	0	0	0	0	0	0	1	0	0	0	0	2	0	0	0	0	0	0	0	1
BVDV1	*Flaviviridae*	*Pestivirus*	1S49	A	0	0	0	0	0	0	0	1	1	0	0	0	1	1	0	0	0	0	0	0	1
HCV1	*Flaviviridae*	*Hepacivirus*	1NB6	A	0	0	0	0	0	0	0	1	1	0	1	0	0	1	0	1	0	0	0	0	1
PolV1	*Picornaviridae*	*Enterovirus*	3OLB	A	0	0	1	0	0	0	0	1	2	0	0	0	1	1	0	2	0	0	0	1	0
HuRV16	*Picornaviridae*	*Enterovirus*	1XR7	A	0	0	1	0	0	0	0	1	2	0	0	0	1	1	0	2	0	0	0	1	0
HuRV1B	*Picornaviridae*	*Enterovirus*	1XR6	A	0	0	1	0	0	0	0	1	2	0	0	0	1	1	0	2	0	0	0	1	0
CoxVB3	*Picornaviridae*	*Enterovirus*	3CDW	A	0	0	1	0	0	0	0	1	1	0	0	0	1	1	0	2	0	0	0	1	0
FMDV	*Picornaviridae*	*Aphthovirus*	2E9Z	A	0	0	1	0	0	0	0	1	2	0	0	0	1	1	0	2	0	0	0	1	0
NORV	*Caliciviriade*	*Norovirus*	3BSO	A	0	0	1	0	0	0	0	1	2	0	0	0	1	1	0	2	0	0	0	1	0
MuNORV1	*Caliciviriade*	*Norovirus*	3UQS	A	0	0	1	0	0	0	0	1	2	0	0	0	1	1	0	1	0	0	0	1	0
RHEV	*Caliciviriade*	*Lagovirus*	1KHV	B	0	0	1	0	0	0	0	1	1	0	1	0	1	1	0	2	0	0	0	1	0
SappV	*Caliciviriade*	*Sapovirus*	2CKW	A	0	0	1	0	0	0	0	1	2	0	1	0	1	1	0	1	0	0	0	1	0
Φ6	*Cystoviridae*	*Cystovirus*	1HI0	P	0	0	0	0	0	2	1	1	1	0	0	0	2	1	0	2	1	0	1	1	2
Qβ	*Leviviridae*	*Allolevivirus*	3AVX	A	0	0	0	1	0	1	1	1	2	0	0	0	1	0	0	1	0	0	1	1	0
IBDV	*Birnaviridae*	*Avibirnavirus*	2PUS	A	0	0	1	1	1	0	0	1	1	0	0	0	0	1	0	2	0	1	0	1	0
IPNV	*Birnaviridae*	*Aquabirnavirus*	2YI9	A	0	0	1	1	1	0	0	1	1	0	0	0	0	1	0	2	0	1	0	1	0
SRV	*Reoviridae*	*Rotavirus*	2R7W	A	0	0	0	0	0	1	2	1	1	0	0	0	0	1	1	2	0	0	1	1	3
MORV3	*Reoviridae*	*Orthoreovirus*	1N35	A	0	0	0	0	0	1	2	1	1	1	1	1	2	1	1	2	0	0	1	1	3
HIV1	*Retroviridae*	*Lentivirus*	3V81	C	1	1	2	1	0	1	2	0	2	2	0	1	0	1	0	1	0	0	1	1	0
HIV2	*Retroviridae*	*Lentivirus*	1MU2	A	1	1	2	1	0	1	2	0	2	2	0	1	0	1	0	1	0	0	1	1	0
MoMLV	*Retroviridae*	*Gammaretrovirus*	1RW3	A	1	1	2	1	0	1	2	0	2	2	0	1	0	1	0	1	0	0	1	1	0

Individual vRdP structures are introduced by PBD code-strain and they are assigned to a virus species. Rows in the matrix represent vRdPs, while the compared features are listed as 21 columns. Compared features are: (A) polymerase product - 0 RNA, 1 DNA; (B) polymerase template - 0 RNA, 1 both DNA and RNA; (C) NA synthesis initiation - 0 *de novo*, 1 protein primer, 2 RNA primer; (D) overall polymerase domain architecture as described in [23] - 0 active site is encircled by finger tips, 1 active site is open (fingers subdomain do not touch thumb subdomain); (E) polymerase core organization - 0 ABC, 1 CAB; (F) motif F length - 0 normal (motif is F2 is present), 1 short (motif F2 is absent), 2 long (insertion is present in motif F); (G) motif F structure - 0 ββα(3_10_)β, 1 βββ, 2 ββ; (H) F - A (C) motif connection - 0 short (≤35 amino acid residues), 1 long structured (>35 amino acid residues); (I) motif A structure - 0 -3_10_, 1 βα, 2 β3_10_; (J) A–B motif connection - 0 ααββ, 1 αββαββ, 2 ββ; (K) length of helix in motif B - 0 normal (≤21 amino acid residues), 1 long (>22 amino acid residues); (L) kink in motif B - 0 absent, 1 present; (M) B - C (D) motifs connection - 0 very short (≤5 amino acid residues), 1 loop (6–14 amino acid residues), 2 long helical (≥15 amino acid residues, at least 8 amino acid residues long helix); (N) motif C length - 0 short (10 amino acid residues), 1 long (>10 amino acid residues); (O) C (B)–D motifs connection - 0 short loop (≤5 amino acid residues), 1 long loop (>5 amino acid residues); (P) motif D structure - 3_10_α-, 1 α-, 2αβ; (Q) position of helix in motif D - 0 normal position, 1 shifted position; (R) D–E motif connection - 0 short (<20 amino acid residues), 1 long structured (<20 amino acid residues); (S) motif E structure - 0 wide, 1 narrow; (T) thumb domain size - 0 large (>180 amino acid residues), 1 small (<180 amino acid residues); (U) priming motif - 0 none, 1 priming loop in thumb subdomain, 2 priming loop in palm subdomain, 3 polymerase C terminal part. Symbols α, β, 3_10_, and L mean α helix, β strand, 3_10_ helix, and loop, respectively.

**Figure 2:**
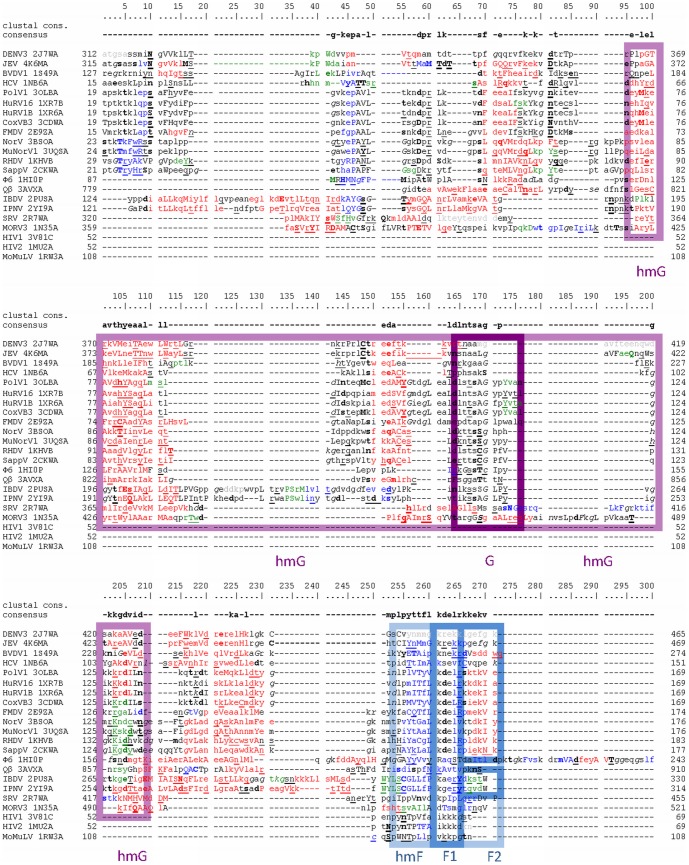
Structure based sequence alignment of vRdPs finger subdomain. vRdPs are listed at the beginning of each row by the name of the virus encoding the appropriate vRdP followed by vRdP PBD code. The number at the beginning and at the end of each row indicates the position of the first and last amino acid residue on the appropriate row in the full-length protein bearing polymerase activity (including all additional protein domains). The numbering above the alignment describes position of individual amino acid residues in the alignment. Amino acid residues forming α helices, 3_10_ helices, and β strands are written by red, green, and blue, respectively. Solvent accessible amino acid residues are written in lower case letters; solvent inaccessible by upper case letters. Amino acid residues with positive phi torsion angle, amino acid residues hydrogen bound to main-chain amide, or amino acid residues hydrogen bound to main-chain carbonyl are underlined, written in bold, or in italic, respectively. Most frequent amino acid residues at each alignment position are listed in a row called consensus. Highly conserved positions (more than 80%) are indicated by uppercase violet letters. The 100% conserved amino acid residues are shown by uppercase red letters. Most upper row shows Clustal calculated consensus. Amino acid residues in conserved sequence motifs G and F typical for all vRdPs are highlighted by violet and dark blue colour frames. Amino acid residues it the conserved structural homomorhps hmG and hmF are highlighted the same but lighter colours.

**Figure 3:**
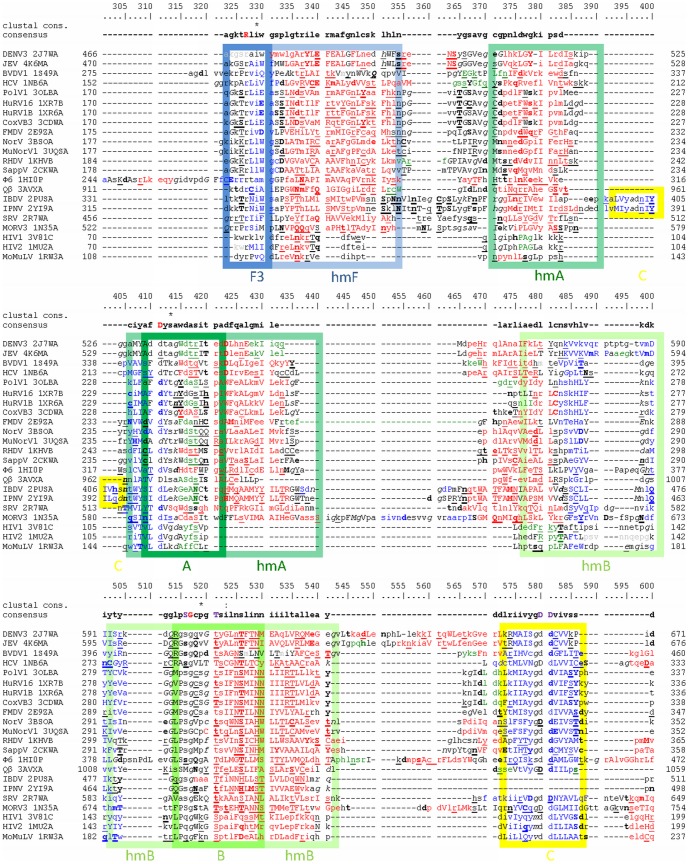
Structure based sequence alignment of vRdPs palm subdomain. Alignment of vRdPs is as in Figure 2. Amino acid residues in conserved sequence motifs F, A, B, and C are highlighted by dark blue, dark green, light green, and yellow frames. Amino acid residues it the conserved structural homomorhps are highlighted the same but lighter colours. The only three 100% conserved amino acid residues in the entire alignment (an arginine residue at position 327 in motif F, an aspartate residue at position 411 in motif, and a glycine residue at position 517 in motif B). The fourth 100% conserved amino acid residue is an aspartate residue in motif C. Despite this aspartate residue is superpostionable in protein structures, it is placed on different position in structure based sequence alignment of protein primary structures thanks to cyclic permutation in IBDV and IPNV RdRPs (see position 397 for birnaviral RdRPs and position 580 for remaining vRdPs).

**Figure 4:**
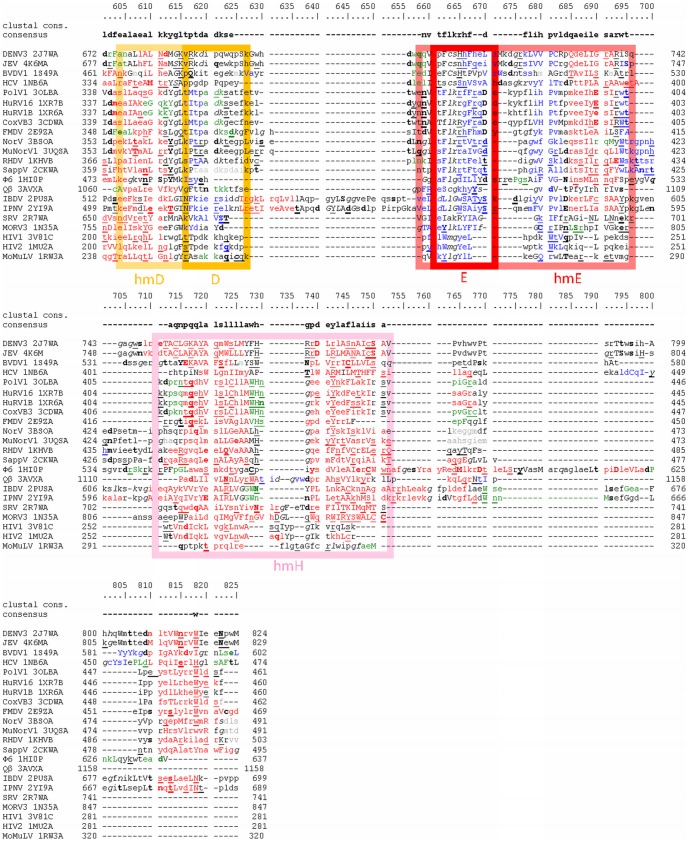
Structure based sequence alignment of vRdPs thumb subdomain. Alignment of vRdPs is as in Figure 2 and 3. Amino acid residues in conserved sequence motifs D and E are highlighted by orange and red frames. Amino acid residues in the conserved structural homomorhps are highlighted the same but lighter colours. hmH homomorph is highlighted in pink.

Automatically created structure based alignment of selected vRdPs including annotated structural features is depicted in Figures 2, 3, and 4.

### Phylogenetic characterization of vRdPs

**Figure 5:**
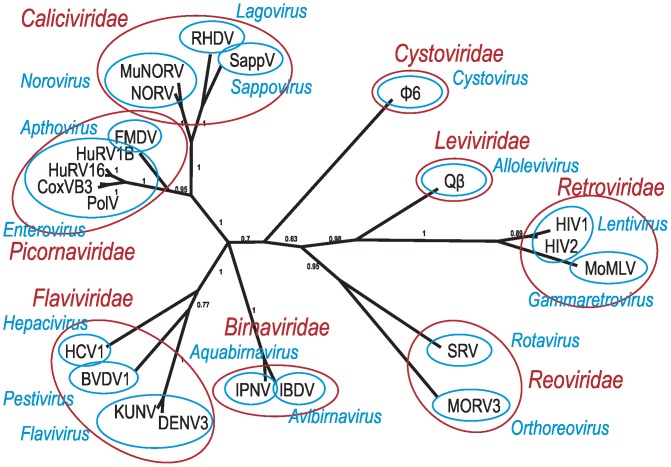
Phylogenetic tree of vRdPs evolution. Phylogenetic tree was calculated by an analysis unifying sequence and structure information. Only names of virus species coding vRdPs are listed in the tree. Individual virus species are grouped in genera (blue) and families (red) according actual ICTV virus taxonomy.

The evolutionary history of vRdPs was reconstructed using the Bayesian clustering analysis. Sequence (structure based sequence alignment) and structural (character matrix) information were used simultaneously in a unified analysis. Combination of these datasets was used to produce a phylogenetic tree with high Bayesian posterior probabilities for most branches (see Figure 5). Despite the high Bayesian support, one polytomy appeared concerning the position of *Birnaviridae* family.

Our phylogenetic analysis classified all vRdPs into groups that correspond to the viral genera and families proposed by ICTV. RdDPs of RNA viruses replicating via DNA intermediate (Baltimore class VI) formed a clearly separated group of vRdPs. The RdRPs of +ssRNA and dsRNA viruses clustered together and did not form any separate groups. This suggests that dsRNA viruses evolved from +ssRNA viruses multiple times, and vice versa. The possible evolutionary scenarios of vRdP evolution and its impact on the reconstruction of RNA virus evolution will be discussed further.

Usage of each data set alone was less statistically powerful than the combined analysis (see Figure S3). Despite, our results rely mostly on sequence information incoming from a structure based sequence alignment. The 21-column character matrix served as a stabilizing element that properly placed ambiguous branches and prevent against long branch artifacts (compare Figure S3 panels A and B and Figure 5).

## Discussion

### Similarities among vRdPs

The vRdPs are an ancient and diversified enzyme group. They share only limited conservation in primary structure, however their protein structure [21], [24] and the mechanism of function [19], [23], [42] are very similar. The vRdPs adopt a conserved right hand conformation with three subdomains termed fingers, palm and thumb. Seven conserved sequence motifs were previously described in vRdPs [16], [17], [37]. Moreover, amino acid residues in these motifs adopt extremely conserved position in vRdPs' [24]. Herein, we described a novel conserved structural motif named homomorph H (hmH) formed by a conserved helix-turn-helix structure in the thumb subdomain of all vRdPs. Despite its high structure conservation, and hmH primary structure is slightly conserved. Function of hmH remains elusive and further biochemical studies will be needed to elucidate it.

Presence of vRdPs in all RNA virus species allows their use in phylogenetic analysis [7], [16], [25]–[31]. This approach was disputed by an extensive study showing the sequence conservation of vRdPs is too low to be successfully and meaningfully used for phylogenetic analysis employing classical methods [32]. The similarities among vRdPs may have evolved by convergent evolution [32], however these conclusions may be challenged by several arguments. 1) The vRdPs share seven conserved sequential collinearly arranged motifs; a phenomenon highly improbable via convergence [16]. 2) The right hand conformation is not the only fold that can be adapted by RNA-dependent polymerases. Cellular RdRPs participating in RNA interference accommodate totally different double barrel conformations [52]. 3) Modern bioinformatics approaches based on Bayesian analyses are more suitable for reconstruction of distant evolutionary relationships [53] than previously described statistical methods [32]. 4) Conserved protein tertiary structure of all vRdPs can supplement missing information in highly diverged protein sequences and allowing us to study the evolution of extremely distantly related proteins [13], [14].

Nevertheless, polymerases can adopt various conformations, changing their topology in response to bound template/incoming nucleotides, steps in polymerization cycle and artificially depending on crystallization conditions. We overcome this by selecting vRdPs' representatives crystallized under similar conditions (see Material and methods).

### How did the vRdPs evolve?

Our phylogram shows the RdDP of *Retroviridae* forms a clearly separate group of RNA viruses replicating via the dsDNA intermediate (Baltimore class VI). This is caused by a series of specific interactions that occurs between template, product and protein, and differs significantly between RdDPs and RdRPs [54]. For example, RdDPs accommodates a conservative aromatic amino acid residue in motif B (alignment position 525 - Figure 3). This position is occupied by aspartate or asparagine interacting with aspartate in motif A (alignment position 416 - Figure 3) in RdRPs discriminating incorporation of dNTPs instead of NTPs [20]. Moreover, the structure of RdDPs is much simpler, many structural motifs are absent, and others are highly reduced [24].

RdRP of the +ssRNA bacteriophage Qβ is the closest relative of retroviral RdDPs. The Qβ polymerase already contains all motifs typical for RdRPs, but is still simpler having no additional structural motifs [55], [56]. As Qβ represents an ancient virus group [57], it is probable that the phylogram may be rooted between Qβ RdRP and retroviral RdRPs.

Rooting the evolutionary tree of vRdPs using cellular right handed polymerases as an outgroup shows, the root is positioned between bacteriophage Qβ RdRP and retroviral RdDPs (Černý et al, under submission). This is in concordance with RNA world theories and theories implicating viruses in the shift from RNA world to DNA world [58].

RdRPs of all RNA viruses are mixed together in our phylogram and they do not follow the Baltimore classification. For example RdRP of +ssRNA Qβ is closely related to the RdRPs of dsRNA viruses than to the RdRPs of other +ssRNA viruses and RdRP of dsRNA birnaviruses tends towards RdRPs of mammalian +ssRNA viruses. The RdRPs can easily replicate both ssRNA and dsRNA without any critical rearrangements in their structure. This is not surprising since picornaviral RdRP were shown to replicate dsRNA even without the aid of a helicase [59].

Primer dependence/independence also apparently evolved multiple times. RdRPs of viruses, which in our phylogram are closer to the expected root (*Leviviridae*, *Reoviridae*, *Cystoviridae*), do not require RNA or protein primer for reaction initialization [60]. This suggests that the original vRdPs were probably primer independent. *De novo* initiation is also typical for many cellular RdRPs [61].

Primer independent RdRPs of viruses from families *Flaviviridae* and *Cystoviridae* share remarkably large thumb subdomains of their RdRPs, allowing accurate positioning of the first incoming nucleotide and RNA polymerization initiation [62]. Despite that both proteins share similar interactions between enzyme, template and incoming nucleotide, the position of the priming motif is different [62].

Viruses from the family *Birnaviridae* and several other families encode cyclic permuted RdRP [31], [37]. It was suggested that birnaviral RdRPs represents an ancient group of polymerases that split from other polymerases before DdDPs, DdRPs, RdDPs and RdRPs were established as four distinct groups [31]. Our results indicate RdRPs with cyclic permutation are younger and they share a common evolutionary ancestor with RdRPs of +ssRNA virus RdRPs.

### What does our model of vRdPs evolution tell us about the evolution of RNA viruses?

Virus evolution is an extremely complicated story. Viral genes and proteins evolve rapidly and relative proteins share only a low degree of homology [3]–[5], making virus phylogenetic reconstruction difficult. It is complicated to generate a proper alignment of selected proteins and the resulting phylograms usually do not have sufficient statistical support [32]. Therefore, a qualitative description of a set of virus features is used for reconstruction of distant phylogenetic virus relationships (capsid architecture, genome replication strategies, etc. [63],[64]). Nevertheless, this approach is sensitive to recombination events between virus and host, or between different viruses, and occurs quite often resulting in a mixture of different genes[65]–[68]. That is why, virus evolution nowadays is not considered as a linear process, but rather as a network [69].

Absence of any universal gene shared by all viruses makes reconstruction of virus evolution even more difficult, despite that some genes are shared among many viruses. An example of such a gene is a jelly-roll capsid protein that is typical for picorna-like viruses (+ssRNA genome), *Microviridae*, *Parvoviridae* (both ssDNA), *Papylomaviridea*, *Polyomaviridae* (both dsDNA), etc. [70], [71]. Jelly-roll capsid protein, however is an inappropriate candidate for a virus phylogenetic marker, since viruses sharing a jelly-roll capsid protein are only distantly related and protein is missing among closely related virus families.

Presence of the vRdPs in all RNA viruses [15] allowed to use the vRdPs as a marker for RNA virus evolution [28]. Nevertheless, their sequence similarity is too low to be used by classical phylogenetic approaches [32]. We overcome this using structure based homology of vRdPs. Our phylogram describing the evolutionary history of vRdPs may be understood as an evolutive phylogram of RNA viruses. Our results are in concordance with the actual concepts of virus evolution [63], [69] and depict the polyphyletic origin of dsRNA viruses. The first group is represented by *Cystoviridae* and *Reoviridae* families, while the second group is represented by the *Birnaviridae* family. *Reoviridae* and *Cystoviridae* share many common features. Both viral groups have similar multilayer capsid organization [72]. They replicate their genome by a conservative manner inside the inner virus capsid [73]. Viruses in *Birnaviridae* family are more similar to +ssRNA viruses. Their cyclically permuted RdRPs are similar to cyclically permuted RdRPs of +ssRNA viruses from *Permutotetraviridae* [31]. Moreover, birnaviruses replicate their genome in a semiconservative manner outside the virus capsid [74] using their guanylylated RdRP as a primer [75] that is similar to protein primed replication of picornavirus-like viruses [76], [77].

Mammalian +ssRNA viruses cluster together forming two monophyletic clades. The first is represented by viruses from the family *Flaviviridae*, while the second by viruses from families *Caliciviridae* and *Picornaviridae*. Regardless that the differences between them are smaller than in the case of dsRNA viruses, both these clades differ in the same biological aspect. Flaviviruses replicates their RNA by a primer independent manner [78], [79]. Their genome is either uncapped [80], [81] or capped by 7-methylguanosine cap [82]. *Caliciviridae* and *Picornaviridae* use vPg protein primer that also caps their genomes [83]. These similarities between mammalian +ssRNA viruses and *Birnaviridae* show they evolved from a common ancestor [31], [70], [84].

The last two groups of RNA viruses, families *Leviviridae* and *Retroviridae*, are distinctly separated. These two groups seem to be extremely ancient and they probably evolved from the last universal common ancestor of all life forms – even before the cell evolution [64], [85], [86]. This is in concordance with recent theories about evolution of ancient life forms, the transition from the RNA into the DNA word and cell evolution [58].

Only a limited number of vRdP protein structures are known now. Nevertheless, they come out from very diverse viral groups that can serve as representatives of other virus groups (*Togaviridae* and *Coronaviridae* would most probably follow *Flaviviridae* etc.). ThevRdPs with known protein structure come from viruses that are usually important as human or veterinary pathogens or represent important biological models. There is no known vRdP protein structure of any plant, protozoan or fungal virus. Moreover, no protein structure of any –ssRNA virus RdRP is known. Since RdRPs of –ssRNA viruses share many sequence motifs with other vRdPs [87]–[89], their structure will most probably be similar to the structure of other RNA viruses. Likewise, vRdPs structures of plant, protozoan and fungal viruses that are often closely related to animal viruses [68] will probably be similar.

## Supporting Information

Figure S1 **Linear organization of protein domains of vRdPs.** The vRdP polymerase finger, palm and thumb subdomains are highlighted by blue, green and red. Remaining protein domains are colored by yellow. Conserved sequential and structural features are not shown. Diagram is in scale.
(TIF)

Figure S2 **Protein structures of all vRdPs involved in analysis.** Molecule positioning is the same as in Figures 1. Polymerase subdomains are highlighted as in the Figure S1: finger subdomain by blue, palm subdomain by green, thumb subdomain by red. Other protein domains are not visible. Molecular rendering in this figure were created with Swiss PDB Viewer.
(PDF)

Figure S3 **Phylogenetic tree of vRdPs evolution based only on sequence or structure data.** Phylogenetic trees were calculated using only sequence (A) or structure (B) borne information. Only names used for virus species coding vRdPs are listed in the tree.
(TIF)

Table S1 **Comparison of hmH and hmE.** The RMSD of hmH and hmE were calculated for all individual couples of vRdPs and compared in table. Individual vRdP structures introduced by PBD code-strain are assigned to virus species. Row E shows RMSD values for hmE. Row H shows adequate values for hmH. It is apparent that RMSD values for hmH are comparable with values for hmE and they are often even lower.
(XLSX)

Text S1 (DOCX)
