# Supplementary figures and images for: Evolution of Tertiary Structure of Viral RNA Dependent Polymerases
Source: PLoS One. 2014 May 9;9(5):e96070. doi: 10.1371/journal.pone.0096070 (PMC4015915; doi:10.1371/journal.pone.0096070)

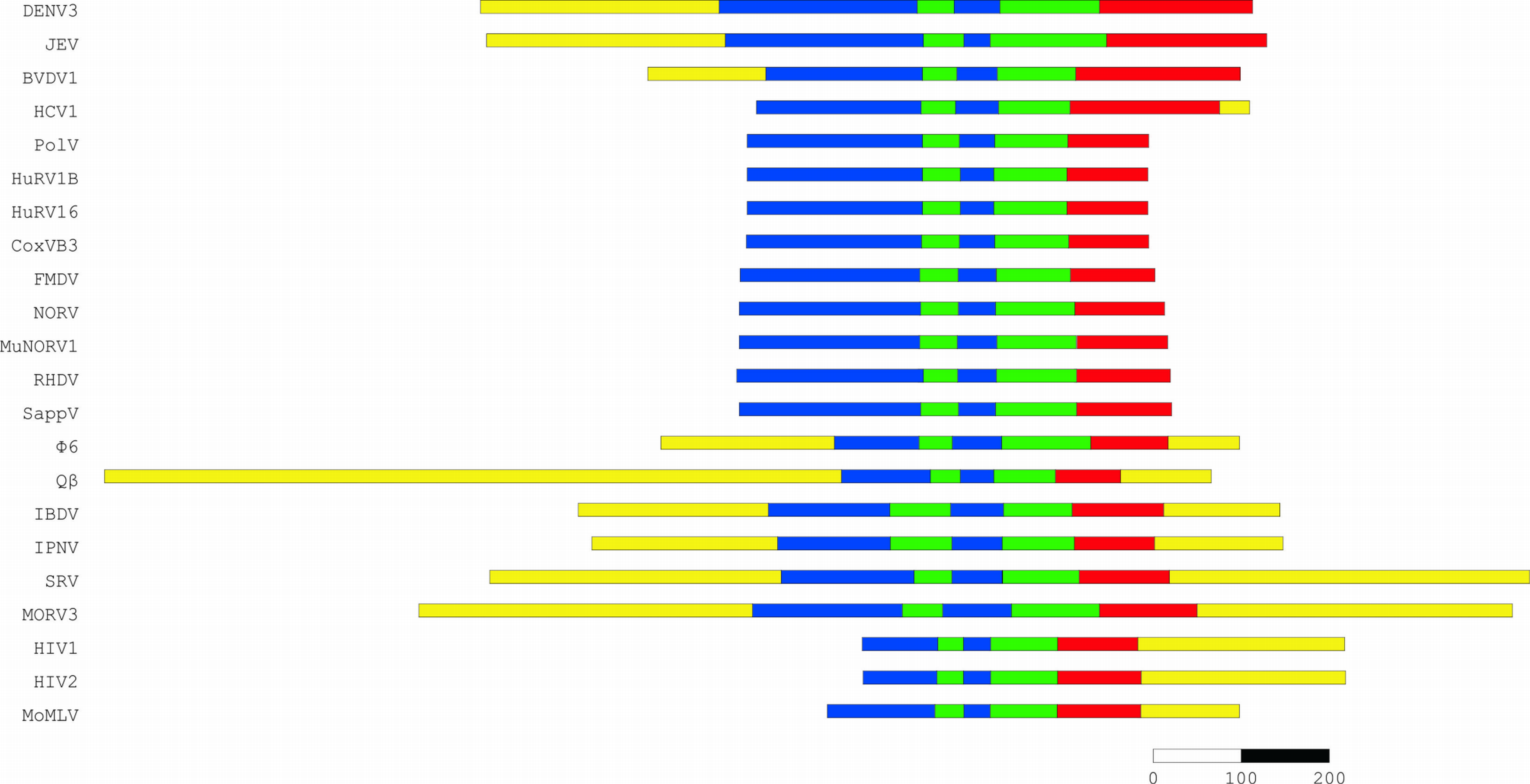

Supplement: Figure S1 — Linear organization of protein domains of vRdPs. The vRdP polymerase finger, palm and thumb subdomains are highlighted by blue, green and red. Remaining protein domains are colored by yellow. Conserved sequential and structural features are not shown. Diagram is in scale. (TIF) [file pone.0096070.s001.tif]

DENV3

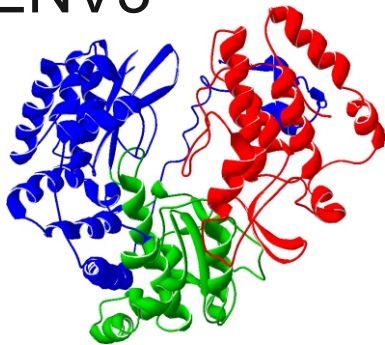

JEV

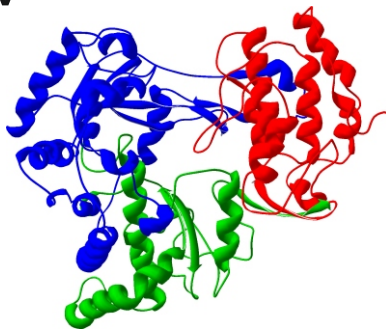

HCV

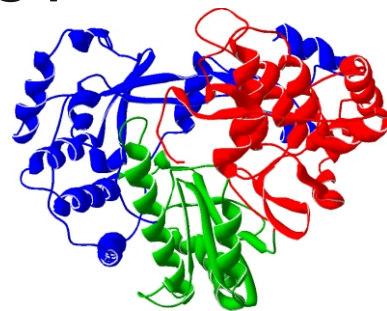

BVDV

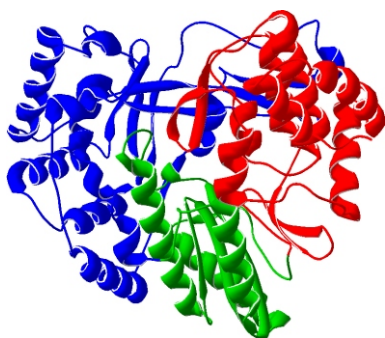

PoIV1

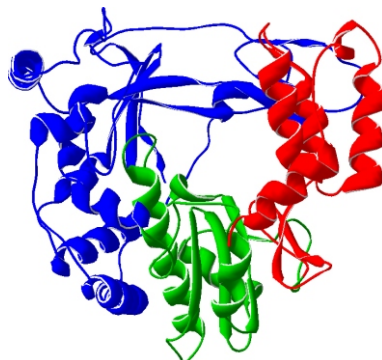

HuRV16

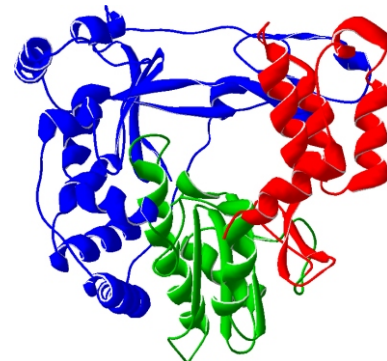

HuRV1B

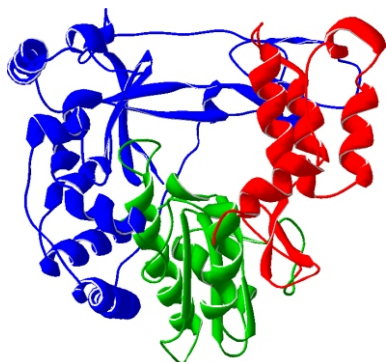

CoxVB3

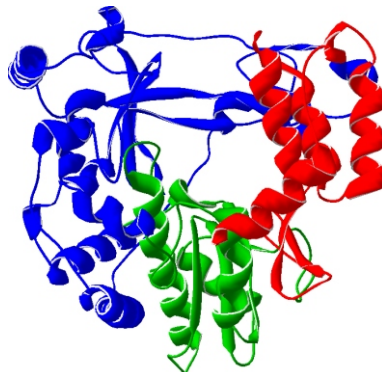

FMDV

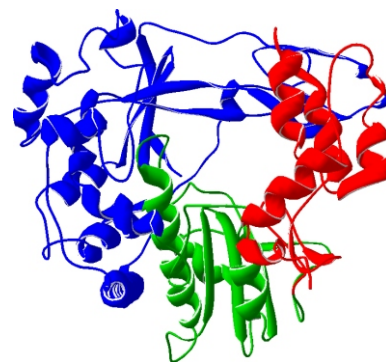

NORV

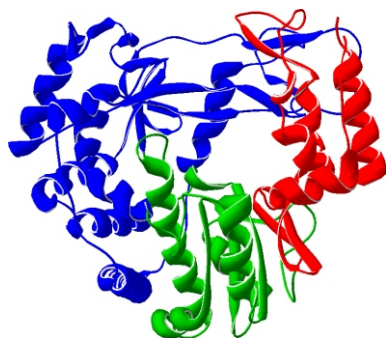

MuNORV1

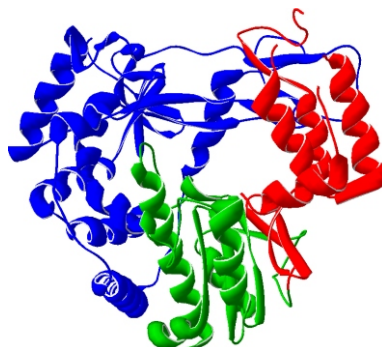

RHDV

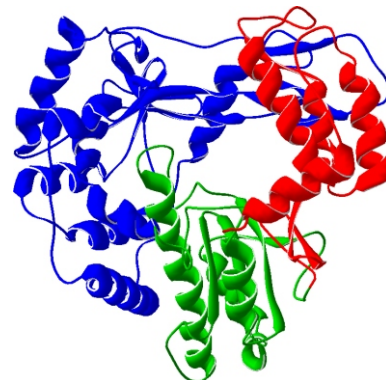

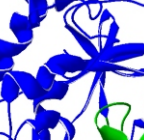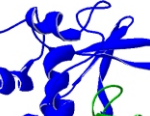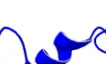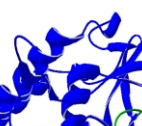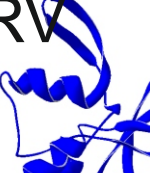

MORV3

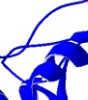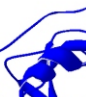

Supplement: Figure S2 — Protein structures of all vRdPs involved in analysis. Molecule positioning is the same as in Figures 1. Polymerase subdomains are highlighted as in the Figure S1: finger subdomain by blue, palm subdomain by green, thumb subdomain by red. Other protein domains are not visible. Molecular rendering in this figure were created with Swiss PDB Viewer. (PDF) [file pone.0096070.s002.pdf]

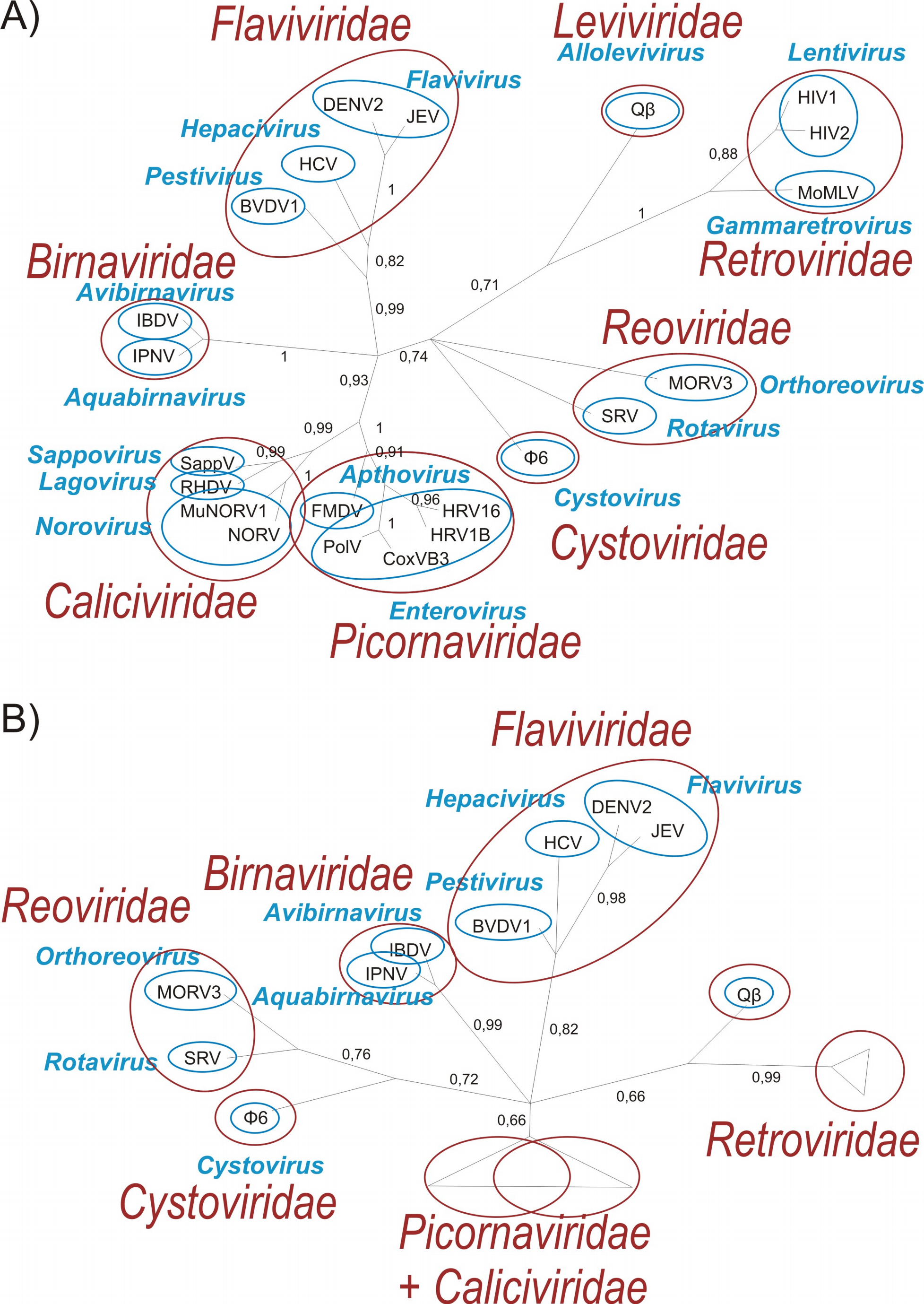

Supplement: Figure S3 — Phylogenetic tree of vRdPs evolution based only on sequence or structure data. Phylogenetic trees were calculated using only sequence (A) or structure (B) borne information. Only names used for virus species coding vRdPs are listed in the tree. (TIF) [file pone.0096070.s003.tif]
