# Supplementary material for: Evolution of Tertiary Structure of Viral RNA Dependent Polymerases
Source: PLoS One. 2014 May 9;9(5):e96070. doi: 10.1371/journal.pone.0096070 (PMC4015915; doi:10.1371/journal.pone.0096070)
Supplement: Text S1 — (DOCX) [file pone.0096070.s005.docx]

**Text S1: Description of vRdPs quantified structural features**

The structural features of vRdPs selected for quantification are as follows:

A) polymerase product

vRdPs can produce either RNA or DNA during virus genome replication *in vivo* [1].

B) polymerase template

Either RNA or both DNA and RNA can be used as a template by selected vRdPs *in vivo* [1].

C) mechanism of polymerization initiation

Three basic types of polymerase reaction initiation are able: *de novo*, protein primed and RNA primed [1]. As in previous cases, some polymerases can use more of them *in vitro* [2], but only the typical case occurring *in vivo* was chosen.

D) overall polymerase domain architecture

We quantified overall polymerase architecture as described in [3]. The polymerases with fully encircled active site (thumbs and fingertips were touching) were marked as closed conformation (typical for most RdRPs). Polymerases where fingers did not touch thumb subdomains were marked as open conformation (see Figures 1).

E) polymerase core organization

The conserved motifs A, B, and C are typically succeeding in alphabetical order. Nevertheless, in birnaviral polymerases the order is cyclically permuted to C, A, and B [4,5] (see Figure 2).

F) motif F length

In most polymerases, motif F consists of three parts motifs F1, F2, and F3. On one hand, some polymerases are missing motif F2 resulting in a shorter version of motif F formed protein loop [6]. On the other hand, as in the case of Φ6 phage, a long insertion is present between motifs F2 and F3 resulting in addition of an extra protein loop (see Figures 1 and 2).

G) motif F structure

Protein loop formed by conserved sequence motif F can adopt three conformations. This conformation can contain two or three β strands. In case it accommodates three β strands, it can either contain or miss a single α (or 3_10_) helix [6] (see Figures 1 and 2).

H) F - A (C) motif connection

This connection is short in case of RdDPs (less than 15 amino acid residues), but significantly longer in case of RdRPs (almost 30 or even more amino acid residues) (see Figure 2).

I) motif A structure

Amino acid residues in motif A are arranged in β strand (sometimes not fully formed) followed by a helix (α of 3_10_). This conformational motif differs among different polymerases [6] (see Figure 2).

J) A - B motif connection

Motifs A and B are divided by a long insertion. Amino acid residues in this insertion can form either two antiparallel β-strands, as in case of RdDPs, or two α helices followed by two β strands, as in case of RdRPs. Polymerases of mammalian orthoreovirus 3 (MORV3) contains an additional insertion of two antiparallel β-strands between the α-helices (see Figure 2).

K) length of helix in motif B

Helix forming motif B is extraordinary long. On average it contains 22 amino acid residues. Shorter helices were termed as short and longer as long for this study (see Figure 2).

L) kink in motif B

Helix forming motif B is straight in RdRPs, while in RdDPs it contains a kink (see Figure 1).

M) B - C (D) motifs connection

Loop following motif B is usually short and unstructured in RdRPs (between 6-14 amino acid residues). On one hand, in some polymerases (for example, in flaviviral polymerases) the connection is longer and accommodates at least 8 amino acid residues in the long helix. On the other hand, in RdDPs the loop is extremely short, being almost reduced (see Figure 2).

N) motif C length

Two antiparallel β strands forming motif C contain, on average, 10 amino acid residues combined. Shorter strands were termed as short and longer as long for this study (see Figure 2).

O) C (B) - D motifs connection

The loop preceding motif D is usually extremely short. Only in reoviral polymerases it contains five or more amino acid residues (see Figure 2).

P) motif D structure

Amino acid residues in motif D are arranged in a helix (α or 3_10_) followed by a β-strand (sometimes not fully formed). The conformation of motif D differs among different polymerases (see Figure 2).

Q) position of helix in motif D

In most polymerases, helix in motif D forms part of a classical RNA recognition motif. Nevertheless, in phage Φ6 polymerase the position of the helix in motif D is skewed [7] (see Figure 1).

R) D - E motif connection

In most vRdPs, the connection between motifs D and E is very short. Only in birnaviral polymerases a long structured motif is inserted (see Figure 2).

S) motif E structure

Motif E serves as an interaction platform between palm and thumb subdomains. In most +ss RNA viruses it folds in three antiparallel β strands forming a wide slightly oblique β sheet. In most dsRNA virus and retrovirus polymerases, however, the sheet is short, straight and much narrower (see Figure 1).

T) thumb domain size

The size of thumb subdomain initiating polymerization reaction *de novo* is much larger compared with the size of thumb subdomain of other polymerases [8] (see Figures 1 and 2).

U) priming motif

Polymerases initiating polymerization reaction *de novo* need a priming motif that serves as a loading platform for the first incoming nucleotide. In flaviviral polymerases, the priming motif is located in the thumb subdomain. In Φ6 polymerase, the priming motif is located in palm subdomain, while in reoviral polymerases the priming motif is situated at the C terminus of the polymerase [3,8] (see Figure 2).

**SUPPLEMENTARY DATA REFERENCES:**

1. Choi KH (2012) Viral polymerases. Adv Exp Med Biol 726: 267-304.

2. Zhong W, Ferrari E, Lesburg CA, Maag D, Ghosh SK, et al. (2000) Template/primer requirements and single nucleotide incorporation by hepatitis C virus nonstructural protein 5B polymerase. J Virol 74: 9134-9143.

3. Ng KK, Arnold JJ, Cameron CE (2008) Structure-function relationships among RNA-dependent RNA polymerases. Curr Top Microbiol Immunol 320: 137-156.

4. Gorbalenya AE, Pringle FM, Zeddam JL, Luke BT, Cameron CE, et al. (2002) The palm subdomain-based active site is internally permuted in viral RNA-dependent RNA polymerases of an ancient lineage. J Mol Biol 324: 47-62.

5. Pan J, Vakharia VN, Tao YJ (2007) The structure of a birnavirus polymerase reveals a distinct active site topology. Proc Natl Acad Sci U S A 104: 7385-7390.

6. Lang DM, Zemla AT, Zhou CL (2013) Highly similar structural frames link the template tunnel and NTP entry tunnel to the exterior surface in RNA-dependent RNA polymerases. Nucleic Acids Res 41: 1464-1482.

7. Butcher SJ, Grimes JM, Makeyev EV, Bamford DH, Stuart DI (2001) A mechanism for initiating RNA-dependent RNA polymerization. Nature 410: 235-240.

8. Ferrer-Orta C, Arias A, Escarmís C, Verdaguer N (2006) A comparison of viral RNA-dependent RNA polymerases. Curr Opin Struct Biol 16: 27-34.
